# Middle Infrared Radiation Induces G_2_/M Cell Cycle Arrest in A549 Lung Cancer Cells

**DOI:** 10.1371/journal.pone.0054117

**Published:** 2013-01-15

**Authors:** Hsin-Yi Chang, Meng-Her Shih, Hsuan-Cheng Huang, Shang-Ru Tsai, Hsueh-Fen Juan, Si-Chen Lee

**Affiliations:** 1 Department of Life Science, Institute of Molecular and Cellular Biology, National Taiwan University, Taipei, Taiwan; 2 Institute of Biomedical Informatics, Center for Systems and Synthetic Biology, National Yang-Ming University, Taipei, Taiwan; 3 Graduate Institute of Biomedical Electronics and Bioinformatics, National Taiwan University, Taipei, Taiwan; 4 Department of Electrical Engineering, National Taiwan University, Taipei, Taiwan; University of Illinois College of Medicine, United States of America

## Abstract

There were studies investigating the effects of broadband infrared radiation (IR) on cancer cell, while the influences of middle-infrared radiation (MIR) are still unknown. In this study, a MIR emitter with emission wavelength band in the 3–5 µm region was developed to irradiate A549 lung adenocarcinoma cells. It was found that MIR exposure inhibited cell proliferation and induced morphological changes by altering the cellular distribution of cytoskeletal components. Using quantitative PCR, we found that MIR promoted the expression levels of ATM (ataxia telangiectasia mutated), ATR (ataxia-telangiectasia and Rad3-related and Rad3-related), TP53 (tumor protein p53), p21 (CDKN1A, cyclin-dependent kinase inhibitor 1A) and GADD45 (growth arrest and DNA-damage inducible), but decreased the expression levels of cyclin B coding genes, CCNB1 and CCNB2, as well as CDK1 (Cyclin-dependent kinase 1). The reduction of protein expression levels of CDC25C, cyclin B1 and the phosphorylation of CDK1 at Thr-161 altogether suggest G_2_/M arrest occurred in A549 cells by MIR. DNA repair foci formation of DNA double-strand breaks (DSB) marker γ-H2AX and sensor 53BP1 was induced by MIR treatment, it implies the MIR induced G_2_/M cell cycle arrest resulted from DSB. This study illustrates a potential role for the use of MIR in lung cancer therapy by initiating DSB and blocking cell cycle progression.

## Introduction

Neoplasm is the major cause of death worldwide, and lung cancer is the leading cause of cancer death [1]. As the smoking habit declines, the incidence of lung cancer has deteriorated in many countries accordingly [2]. Except smoking, other factors such as asbestos, radon or heavy metals exposures also contribute to lung cancer [3]. Statistics data from a 2008 International Agency for Research on Cancer (IARC) risk assessment indicates that lung cancer kills about 1.4 million people per year globally [4]. Due to the high occurrence and lethality of lung cancer, the related therapy is progressing to solve these problems.

The electromagnetic radiation from solar radiation can be divided into several regions, including γ-ray, x-ray, ultraviolet (UV), visible light, infrared (IR) radiation, microwave and radio wave. Among the solar radiation, IR light with wavelengths ranging from 0.76–1000 µm can be divided into three regions, near-infrared (NIR, 0.76–1.5 µm), middle-infrared (MIR, 1.5–5.6 µm) and far-infrared (FIR, 5.6–1000 µm). Applications of IR have been widely established in daily uses and clinical purposes, including cutaneous microcirculation, wound healing, gas sensors, and remote temperature measurement [5].

Previous studies have indicated that NIR triggered a retrograde mitochondrial signaling response resulting ROS mediated matrix metalloproteinase-1 (MMP-1) production via mitogen-activated protein kinases (MAPKs) pathway [6]. It also showed that NIR protected human dermal fibroblast from UV-induced cytotoxicity by inducing Hsp27 which prevents apoptosome assembly, an initial event of apoptosis [7], [8]. *In vivo* studies demonstrated that NIR increased the angiogenic inducer vascular endothelial growth factor and decreased the angiogenic inhibitor thrombospondin-2, initiating dermal angiogenesis in human skin [9]. Moreover, exposure of FIR promoted angiogenesis in human microvascular endothelial cells along with the activation of p38 and extracellular signal regulated kinase signaling [10], enhanced blood circulation in the skin [11], [12] and exerted anti-inflammatory activity in vascular endothelium [13]. It also reported that FIR inhibited cell proliferation through enhancing the expression of cyclic AMP-dependent transcription factor (ATF) 3 in cancer cells whose basal expression level of heat shock protein (HSP) 70A were low [14], [15].

The effects of MIR on cancer cells, however, remain unknown. This study aimed to investigate the effects of MIR with wavelength band in the 3–5 µm regimes on the highly proliferated cancer cells. To this end, we developed an MIR emitter and constrained the MIR wavelength at 3 to 5 µm. Since the molecular C-H, N-H and O-H bonds can be excited to generate stretching vibrations by 3–5 µm infrared, it is expected that the important biochemical reaction will be affected by the irradiation of infrared with wavelength in this range [16]. We revealed that MIR reduced cell viability, caused significant changes in cytoskeleton arrangement, and induced G_2_/M cell cycle arrest which might be contributed by induction of double-strands breaks (DSB) in DNA along the ATM/ATR-p53-p21 axis.

## Results

### The Wavelength of MIR was Constrained at 3–5 µm and the Temperature of Culture Medium was Consistent at 37°C

**Figure 1:**
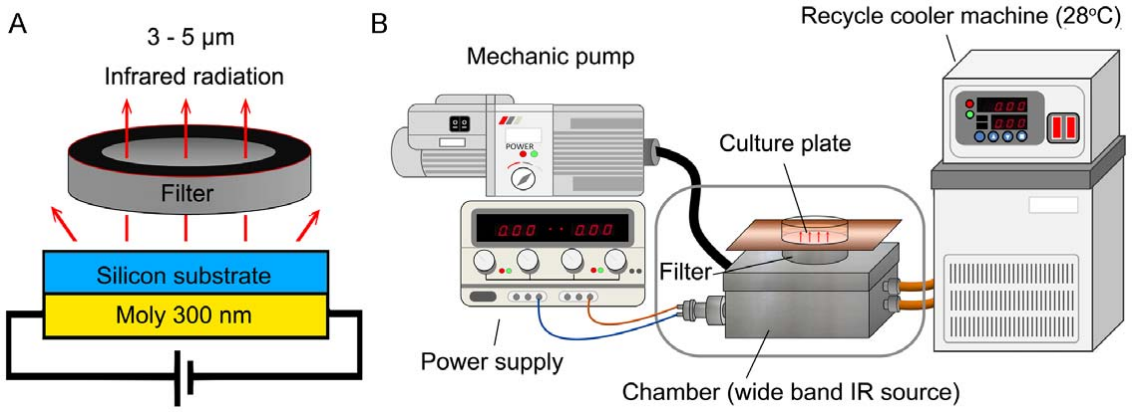
The MIR emitter. (A) The side view of wide band blackbody source. (B) Schematic diagram showing the setup for the MIR irradiation experiment. The cells were plated onto 12-well plates and cultured in an incubator with 100% humidity, at 37°C and with 5% CO_2_.

The wide band blackbody source was fabricated to provide broad band MIR and set in a metal chamber to avoid the disturbance from environment (Figure 1). With the increasing of heating temperature, the emission power of silicon substrate was elevated correspondingly. The radiation intensity was set to 3 mW/cm^2^ by adjusting the heating temperature and measuring the magnitude by THORLAB PM100D power meter. To remove the heat effects of MIR, we set the recycle cooler machine at 28°C to cool the air in the chamber where provided the MIR source thus maintain the temperature of culture medium at 37°C. The arrangement of the apparatus is shown in Figure 1B.

### The Cell Proliferation of A549 Cells Remained Unchanged in the MIR Exposed Medium

To distinguish whether the effects of MIR occurred directly on cultured cells or indirectly through altering the cell medium, the culture medium was exposed to MIR for 48 hours prior to its addition to the cell culture. After A549 cells (2×10^4^ cells per well) were seeded onto a 12-well plate, we substituted the culture medium with MIR-exposed or unexposed medium for a further 48 hours and then determined cell viability by MTT assay. We observed that cell proliferation of A549 cells was not significantly altered under MIR-exposed medium compared to unexposed medium (Figure S1). From here, we applied a 48-hour period of exposure to the cells for all experiments unless otherwise specified.

### MIR Inhibited the Cell Proliferation and Altered the Morphology of A549 Cells

**Figure 2:**
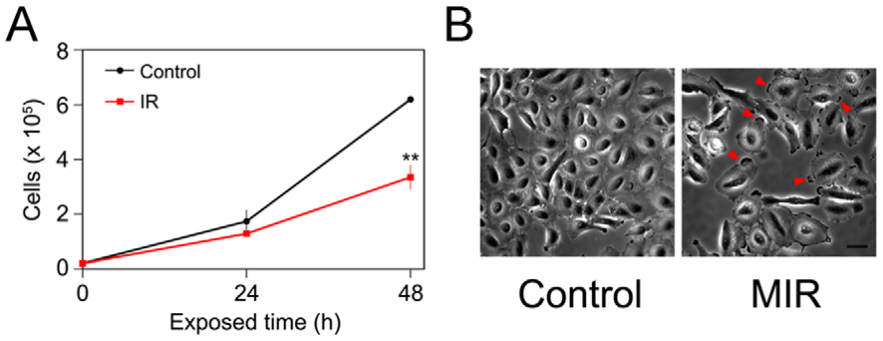
Effects of MIR exposure on cell proliferation and the morphology of A549 cells. (A) The cell numbers were measured at 0, 24 and 48 hours after MIR exposure by using Trypan blue and a hemocytometer. The average cell numbers of control (unexposed) and MIR exposure treatments were expressed as means ± SD from three independent experiments. ** *P*<0.01. (B) Cell images were observed by phase-contrast microscopy after 48 hours MIR exposure. Arrows indicate the radial aprons of enlarged cells. Scale bar represents 50 µm.

To investigate the effect of MIR on cancer cells, lung adenocarcinoma cells A549 were utilized to assess the cytotoxicity of MIR and the normal lung fibroblasts MRC-5 were tested for comparison. Cells (2×10^4^) were plated in 12-well culture plates overnight prior to MIR exposure. The cell viability was determined by MTT assay and trypan blue based cell counting after MIR exposure. The results indicated that the proliferation of A549 cells was significantly suppressed by MIR exposure for 48 hours (Figure 2A), while the growth and morphology of MRC-5 cells were not affected by MIR treatment (Figure S2A, S2B). Interestingly, we revealed morphological changes to the A549 cells upon MIR exposure. We observed that MIR-exposed A549 cells were more rounded in shape, enlarged in size, and formed a radial apron under phase-contrast microscopic examination (Figure 2B). The results imply that MIR might regulate the cytoskeleton dynamics which determines the cell morphology.

### MIR Exposure Activated the Reorganization of Actin Filament, Vinculin and Microtubule

**Figure 3:**
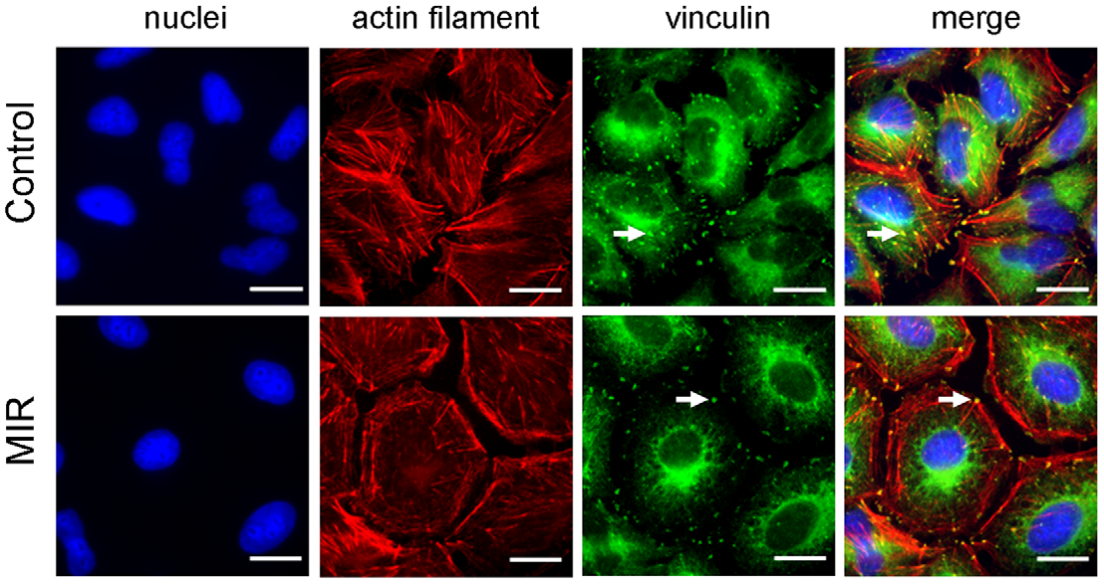
Effect of MIR exposure on the actin filaments and focal adhesions of A549 cells. Cells were seeded onto glass coverslips in 12-well plates, exposed to MIR for 48 hours, fixed for staining and visualized by fluorescence microscopy. Actin filaments were tagged with rhodamine-labeled phalloidin (red), vinculin was labeled with mouse anti-vinculin antibody and the corresponding FITC– conjugated secondary anti-mouse IgG antibody (green), and nuclei were stained with DAPI (blue). Scale bar represents 10 µm. Arrows indicate the position of vinculin.

**Figure 4:**
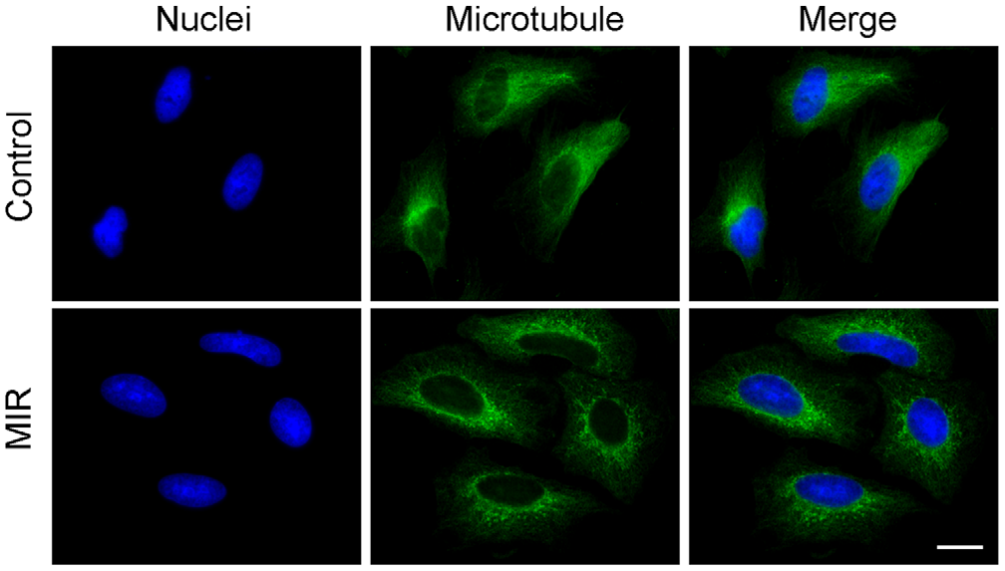
Effect of MIR exposure on the microtubule networks of A549 cells. Cells were seeded onto glass coverslips in 12-well plates, exposed to MIR for 48 hours, fixed for staining and visualized by fluorescence microscopy. Microtubules were labeled with α–tubulin antibody and the corresponding FITC–conjugated secondary antibody (green), and nuclei were labeled with DAPI (blue). Scale bar represents 10 µm.

The cytoskeleton plays an important role in regulating cell shape [17], [18], and both actin filaments and microtubules are known to affect the formation and distribution of cell focal adhesions [17] which determine cell morphology and motility. To distinguish the effects of MIR on cytoskeleton, we performed immunofluorescence staining to examine whether the two important components of cytoskeleton, actin filaments and microtubules, as well as the focal adhesion molecule vinculin involved in this morphological change. The results showed that MIR induced a significant decrease in F-actin containing stress fibers as determined by staining with rhodamine-labeled phalloidin (Figure 3). Furthermore, the actin filaments exhibited a dense meshwork of unpolarized arrangement and the vinculin was aggregated around the cell periphery in MIR-exposed cells (Figure 3), implying that MIR may inhibit cell migration by regulating the reorganization of actin filaments and distribution of vinculin [19]. On the other hand, microtubules were distributed at the perinuclear cytoplasmic regions after MIR treatment (Figure 4). This specific distribution of irregular microtubule fragments suggests that MIR could cause G_2_/M cell cycle arrest as previously demonstrated [20], [21].

### MIR Exposure Regulated the mRNA Expression Level of G_2_/M Regulated Genes in A549 Cells

**Figure 5:**
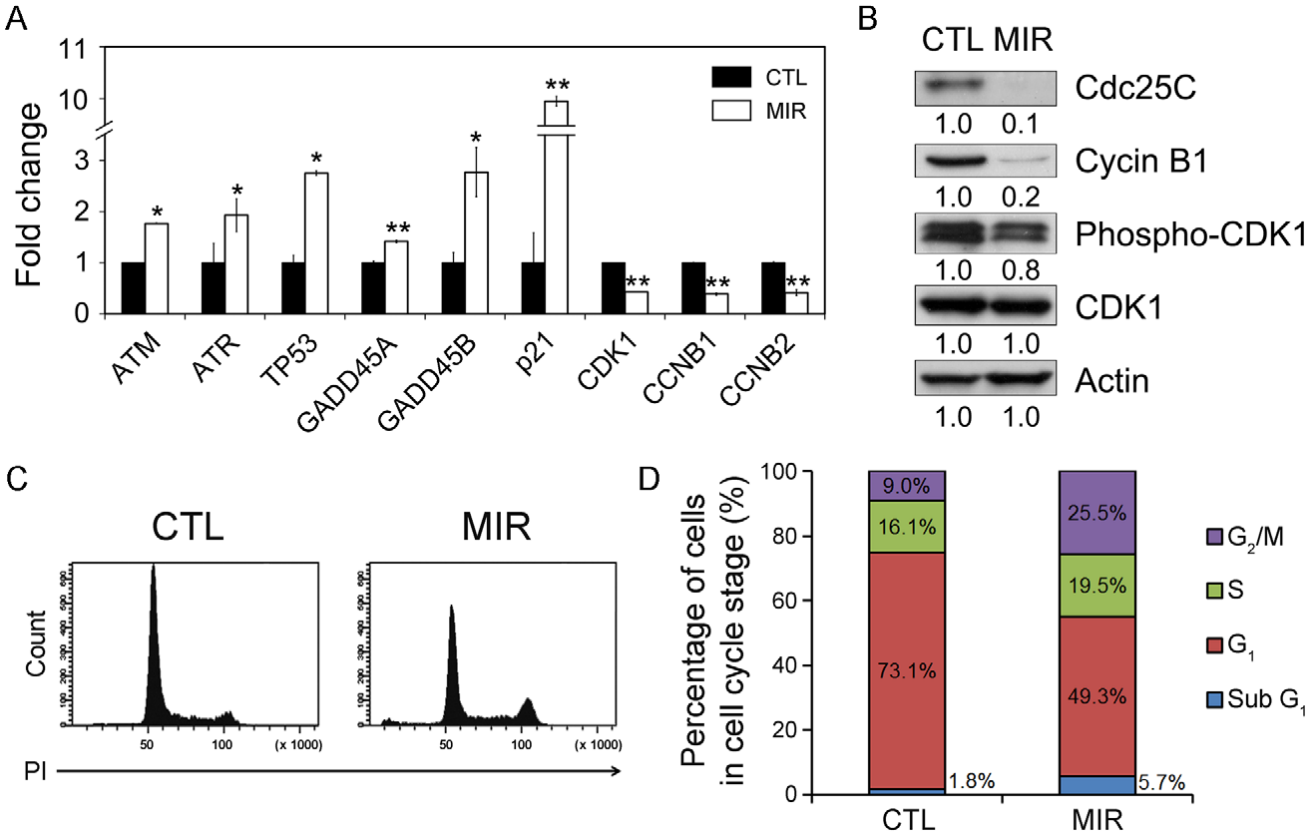
MIR exposure induced G_2_/M cell cycle arrest in A549 cells. Cells were exposed to MIR for 48 h, and harvested for RNA and protein extraction. (A) Gene expression of genes involved in regulation of G_2_/M transition (x-axis). The y-axis indicates the relative transcript quantities calculated using the ΔΔCt method with GAPDH as the reference gene amplified from each sample. The data are presented as mean ± S.D. (*n* = 3). * *P*<0.05, ** *P*<0.001. (B) Protein expression levels were examined by Western blot with actin as the internal control. All experiments were repeated three times. (C) Flow cytometric analysis of DNA content. Cells were exposed to MIR for 48 h. Cells from six independent experiments were collected for analyzing cell cycle distribution. (D) The percentage of cells in each phase was obtained by MultiCycle analysis.

Since the distribution of cytoskeleton imply a possible role of MIR in regulating cell cycle progression, it is critical to examine the expression of genes involved in G_2_-M transition were further selected to be validated. The G_2_/M cell cycle checkpoint responds to DNA damage and involves the activation of ataxia-telangiectasia mutated (ATM) and ataxia-telangiectasia and Rad3-related (ATR) proteins [22]. Both ATM and ATR activate p53 by phosphorylation of Ser15 in response to DNA damage, thus increasing the transcription of growth arrest and DNA damage inducible gene (GADD45) and p21, which are required for inhibiting expression of the key regulators of the G_2_/M transition, cyclin-dependent kinase 1 (CDK1) and cyclin B [23], [24], [25], [26]. The expression of genes involved in inducing G_2_/M arrest were increased in MIR-treated A549 cells, including ATM, ATR, p53, GADD45A, GADD45B, and p21 (Figure 5A). In contrast, the mRNA expression levels of CDK1, CCNB1 and CCNB2 were decreased after MIR exposure (Figure 5A). The results demonstrate that MIR exposure activates the expressions of ATM, ATR, p53, and p21 genes in response to DNA damage and regulates the genes to control G_2_/M cell cycle progression.

### MIR Exposure Abolished the Expression of Cdc25C and Cyclin B1, and Decreased the Phosphorylation of CDK1

The cell cycle progression from the G_2_ to M phase is regulated by activation of CDK1, whose activity is dependent upon coordination with cyclin B [27], [28]. The activation of the CDK1/cyclin B complex is maintained through phosphorylation at Thr161 and dephosphorylation at Thr14 and Tyr15 of CDK1 [27], [28]. Dephosphorylation of the Thr14 and Tyr15 residues in CDK1 is catalyzed by phosphatase Cdc25C. It is thought of as a rate-limiting step for G_2_ entry into mitosis [27], [29]. Considering the role of the CDK1/cyclin B complex and Cdc25C in regulating G_2_ to M phase transition, we assessed whether MIR exposure altered the protein expression of CDK1, cyclin B1, and Cdc25C, as well as the phosphorylation of CDK1. The results showed that the phosphorylation of CDK1 protein at Thr161 and the levels of cyclin B1 and Cdc25C were all reduced in cells treated with MIR (Figure 5B). It indicates that MIR exposure induced a typical G_2_/M cell cycle arrest in A549 cells by regulating cyclin B1 and Cdc25C expression, and CDK1 phosphorylation.

### MIR Exposure Resulted in Cell Cycle Arrest at G_2_/M Phase

We next examined whether the cell cycle distribution of A549 were affected by MIR irradiation. To obtain the DNA content, we performed flow cytometry to analyze PI-labeled cells. The results showed that the cells in G_2_/M population were increased under MIR exposure. Coordinately, the G_2_/M regulators were both activated in their transcriptional, translational and post-translational levels, leading to the accumulation of cells in G_2_/M phase.

### MIR Exposure Triggered Colocalization of 53BP1 and γ-H2AX Nuclear Foci in Response to Reactive Oxygen Species (ROS) Mediated DNA Damage

**Figure 6:**
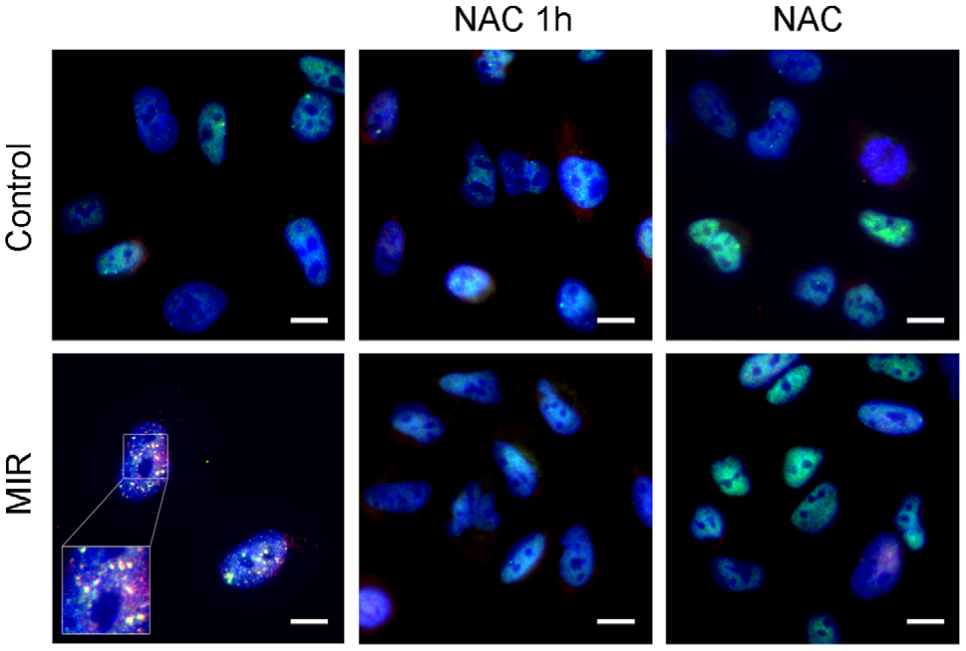
Effect of MIR exposure on DNA double strain breaks in A549 cells. Cells were seeded onto the glass coverslip in 12-well plate, exposure by MIR for 48 hours in the presence or absence of 10 mM N-Acetyl-Cysteine (NAC). Cells were treated with NAC for 1 h prior to MIR exposure (NAC 1 h) or cotreated throughout the exposure for 48 h (NAC). Cells were fixed for staining and visualized by fluorescence microscopy. 53BP1 was labeled with rabbit anti-53BP1 antibody and corresponded FITC–conjugated anti-rabbit IgG antibody (green), γ-H2AX was labeled with mouse anti-γ-H2AX antibody following corresponded PE–conjugated anti-mouse IgG antibody (red), and nuclei were labeled with DAPI (blue). Scale bar represents 10 µm.

Since MIR activated the ATM/ATR-p53-p21 axis which is the DNA damage checkpoint pathway, it is critical to investigate whether the MIR caused DNA damage in A549 cells. Previous studies showed that tumor suppressor p53 binding protein (53BP1) and γ-H2AX participate in the ATM-dependent DNA damage-signaling pathway and form nuclear foci in response to ionizing radiation caused DNA damage [30], [31]. To examine this, A549 cells were fixed with acetone and stained for 53BP1 and γ-H2AX after MIR exposure. We exhibited that 53BP1 (Figure S3A) and γ-H2AX (Figure S3B) were dispersedly localized in the nuclei of unexposed cells, but formed numerous distinguished subnuclear foci in response to MIR. We also demonstrated that the 53BP1 and γ-H2AX foci were colocalized in nucei of MIR exposed cells. The formation and the colocalization of 53BP1/γ-H2AX foci were diminished upon pretreatment or cotreatment of 10 mM ROS scavenger NAC (Figure 6). We can postulate that MIR induced G_2_/M cell cycle arrest might result from ROS mediated DNA damage of which the damage markers 53BP1 and γ-H2AX foci were observed in this study.

## Discussion

Because the molecular alterations involved in regulating cell cycle progression are accompanied by a failure to arrest proliferation in cancer cells, inhibition of cell proliferation by interfering with the cell cycle is a promising approach for anticancer therapy. In this study, we observed that MIR exposure can inhibit the proliferation of A549 cells (Figure 2A), and also lead to an enlarged, radial apron and rounded shape cell morphology (Figure 2B) by affecting the arrangement and distribution of actin filaments (Figure 3), focal adhesion (Figure 3), and microtubules (Figure 4). The perinuclear fragmented distribution of microtubules is consistent with the cell morphology observed during G_2_/M cell cycle arrest, which was further validated by analyzing cell cycle distribution, the gene expression and protein regulation of regulators of the G_2_/M check point (Figure 5). In advanced, we also demonstrated that MIR could induce formation of and colocalization 53BP1 and γ-H2AX nuclear foci (Figure 6) in response to DNA damage which typically activates ATM/ATR-p53-p21 pathway resulting G_2_/M cell cycle arrest.

MIR is the part of the light from solar radiation which can cause DNA damage by direct excitation of DNA or indirect mechanism that involves excitation of other cellular chromophores [32]. The direct excitation process is caused by short wavelength radiation, such as UVB (280–315 nm) and UVC (100–280 nm) and mostly leads to generate cyclobutane pyrimidine dimers and photoproducts, which are produced by absorption of radiation by DNA [32]. On the other hand, the indirect mechanism is contributed by reactive oxygen species (ROS) which is generated by endogenous photosensitizers. The indirect DNA damage is caused by longer wavelength radiation above 320 nm, such as UVA (315–400 nm) and near-visible light, at which DNA absorbs only weakly [33], [34]. Radiation with longer wavelength thus is absorbed by photosensitizers to generate ROS. After UVA light absorption, endogenous photosensitizer cross over to a triplet state and transfer energy to generate singlet oxygen [35]. These UVA irradiated photosensitizers include flavins [36], NADH/NADPH [37], urocanic acid [38] and some sterols [39]. Because of the short half time in cells, the singlet oxygen is only present after radiation [40]. However, ROS can be presented for an extended period after radiation exposure since the additional ROS can be produced by initial species [41]. The superoxide anion radical (^•^O_2_^−^), hydrogen peroxide (H_2_O_2_), and hydroxyl radical (^•^OH) are belonged to ROS group, all of which can be generated by endogenous mechanism as by-products of normal mitochondrial activity or exogenous stress [42].

Once the exogenous stress induced ROS level are dramatically higher than the cell can eliminate, oxidative stress occurs and results in oxidative DNA damage by DNA protein crosslink, base and sugar modification, depurination or deprimidination [43], [44], [45], [46], [47]. The oxidative DNA damage induced by ROS can trigger cell cycle checkpoint responses including recruitment of 53BP1 and γ-H2AX followed by degradation of CDC25C for G_2_/M arrest as we observed, thus provides additional time for DNA repair [48], [49]. Moreover, NIR have been found to generate ROS derived from mitochondria, and cytochrome *c* oxidase have been suggested as a possible photoreceptor [6], [50]. The evidences suggest that IR could accelerate the oxidative phosphorylation reaction in mitochondria by irradiating photoreceptors such as cytochrome c oxidatse and NADH. The enhanced rate in oxidative phosphorylation generates higher ROS thus contributes to indirect damages in DNA.

In this study, we found that MIR exposure suppressed the proteins level of CDC25C and cyclin B1, and inhibited the phosphorylation of CDK1. Downregulation of CDC25C would block the activation of CDK1, resulting in dissociation of cyclin B1 and prevention of cell cycle progression from G_2_ to M phase. Furthermore, we exhibited that 53BP1 andγ-H2AX form numerous subnuclear foci in response to MIR treatment. 53BP1 takes part in the ATM-dependent DNA damage-signaling pathway and forms nuclear foci in response to ionizing radiation caused DNA damage [30], [31], while γ-H2AX facilitates the recruitment of a number of damage response proteins, such as BRCA1, MDC1 and RAD51 for DNA repairing [51], [52]. It is possible that MIR exposure induced G_2_/M arrest is caused by DNA damage, even though the wavelength of MIR is close to NIR which is hard to cause direct damage in DNA. Here, we postulate that MIR exposure may be absorbed by endogenous photosensitizer thus elevating ROS and causing oxidative DNA damage.

Previous studies showed that hydrogen peroxide induced G_2_/M cell cycle arrest and influenced the organization of microtubules and actin filaments relay on the capacity for reducing the oxidized cytoskeleton proteins or balance of thiol disruption [51], [52], [53]. Hydrogen peroxide increased monomeric tubulin, but decreased the polymerized tubulin suggesting hydrogen peroxide caused depolymerization of microtubules. In this study, MIR exposure caused the perinuclear distribution and densely aggregation of microtubules thus decreasing the microtubule networks. This observation is similar to microtubule targeting drugs, such as Vinca alkaloids [54]. The perinuclear distribution of microtubules implies that MIR exposure might induce oxidative stress thus disturbing microtubules networks.

In this study, we found that MIR exposure induced DNA damage response. Previous studies showed that γ-H2AX nuclear foci was found to be colocalized with repair proteins involved in homology recombination and nonhomologousend-joining (NHEJ) [55]. Other repair proteins, including MCD1/NFBD1 and 53BP1, were also documented to interact with γ-H2AX to form nuclear foci [56]. Previous reports demonstrate that conformational changes in 53BP1 is caused by phosphorylation of 53BP1 by ATM thus exposing the chromatin-binding domain which participates directly in repair of DNA DSBs by activating DNA ligase IV/Xrcc4 complex in NHEJ pathway [57]. In this study, we found that MIR exposure formed nuclear foci of 53BP1 and γ-H2AX (Figure 6) implies that MIR exposure induce DNA repair in response to DAN damage.

In summary, this study exhibit MIR of the wavelength in 3–5 µm can alter the organization of actin filament, microtubule and vinculin, and cause inhibition on cell cycle progression through activating ATM/ATR-p53-p21 axis in response to DNA damage, also the Cdc25C regulating pathway in parallel thus resulting in downregulation of dephosphorylation in CDK1 and cyclin B. In particular, our study shows the first evidence on the inhibitory effect of MIR in lung cancer cells and provides useful information for cancer therapy.

## Materials and Methods

### Middle Infrared Radiation (MIR) Emitter

The wavelength of MIR generated from the wide band blackbody source was limited in the range between 3 to 5 µm by using a 3–5 µm band pass sapphire wafer (SingHuang Technology Co., Ltd., Taipei, Taiwan) (Figure 1A). The wide band pass filter was designed to isolate 3–5 µm atmospheric windows with a diameter of 25.4 mm ±0.1%. The 50% cut on/cut off transmission points were set at 3.0 µm ±4% and 5.0 µm ±4%, respectively. The filter exhibited average transmission in the pass band higher than 60% and less than 0.1% transmission levels outside the pass band. A 300 nm thick molybdenum film was deposited by plasma deposition on the back side of an n-type silicon substrate as a heating source (Figure 1A). The radiation intensity was measured by THORLAB PM100D power meter to be 3 mW/cm^2^. To maintain the cell culture temperature, recycle cooler machine was set to maintain the temperature of culture medium at 37°C as shown in Figure 1B. Cells seeded onto 12 well tissue culture plates (Corning Costar, Corning, NY, USA) before 24 h of IR exposure were placed on the filter as indicated in Figure 1B.

### Cell Culture

Human lung epithelial cells A549 (ATCC, CCL-185) and human fetal lung fibroblast cells MRC5 (ATCC, CCL-171) were obtained from American Type Culture Collection (Manassas, VA, USA). The A549 cells were cultured in Dulbecco’s modified Eagle’s medium (DMEM, Gibco, Grand Island, NY, USA) supplemented with 10% fetal bovine serum (Biological Industries, Beit Haemek, Israel). The MRC5 cells were cultured in Minimum Essential Medium (Gibco) supplemented with 1 mM sodium pyruvate (Gibco), 0.1 mM non-essential amino acid (Gibco), and 10% fetal bovine serum (Gibco). Both the cell lines were free from mycoplasma as detected by PCR based method and cultured at 37°C with 5% CO_2_.

### Immunofluorescence Staining

A549 cells were seeded onto the glass coverslip in 12-well culture plate, cultured for 1 day, and exposed by MIR or cultured in the dark for further 48 h. Cells were fixed and permeabilized with precool acetone for 5 min at −20°C and then incubated with 1% BSA (Bioshop, Burlington, ON, Canada) in PBS as blocking buffer for 30 min at room temperature. Subsequently, cells were labeled with mouse monoclonal anti-tubulin antibody (Millipore, Billerica, MA, USA; 1∶1000), mouse monoclonal anti-vinculin antibody (Millipore; 1∶200), rabbit polyclonal anti-53BP1 antibody (GeneTex, San Antonio, TX, USA; 1∶500), or mouse anti-γ-H2AX antibody (Abcam, Cambridge, MA, USA; 1∶500) at 4^o^C overnight. After washed with PBST (PBS containing 0.05% Tween-20, Sigma-Aldrich, St Louis, MO, USA) three times, cells were labeled with corresponding secondary anti-mouse IgG-Alexa 488 (Invitrogen, Carlsbad, CA; 1∶1000), anti-mouse IgG-PE (Abcam; 1∶1000), or secondary anti-rabbit FITC-IgG (Millipore; 1∶200) for 1 hour in the dark at room temperature. Cells labeled with anti-vinculin were counter stained with TRITC-conjugated Phalloidin (Millipore; 1∶1000) for 15 min in the dark at room temperature. The cells were then washed with PBST three times and mounted with ProLong® Gold reagent with DAPI (Invitrogen). Images were acquired by fluorescence microscope with Leica HCX FL PLAN 100×/1.25 oil objective using a SPOT camera (Diagnostic Instruments, Sterling Heights, MI, USA) and SPOT Advanced software (Diagnostic Instruments).

### Cell Viability Assay

2×10^4^ cells were seeded on 12-well plates per well and allowed to attach overnight before MIR exposure. The MTT powder (3[4,5-dimethylthiazol-2-yl)-2,5-diphenyltetrazolium bromide, Sigma-Aldrich) were prepared as stock in PBS at the concentration of 5 mg/ml and filtered. After exposed by MIR for 48 h, 150 µl MTT solution was added to each well. The cells were incubated at 37°C for 3 hours until the violate crystal formed. Formazan crystals were dissolved with 500 µl dimethyl sulfoxide (DMSO, Scharlau Chemie, Barcelona, Spain) and agitated avoided from light for 15 min to completely solubilize the crystals. The absorbance was measured at 570 nm on an ELISA reader (BioRad Laboratories, Hercules, CA, USA). All experiments were repeated three times.

### RNA Extraction and Reverse Transcription

Total RNA was isolated using TRIzol reagent (Invotrogen) after 48-h exposure. In brief, 400 µL TRIzol reagent (Invitrogen) was added to each well of 12-well TC plate once the medium was removed. Samples were collected from three independent experiments and stored at −80^o^C. The RNA extraction was performed according to the manual, and the RNA concentration and quality were determined by NanoDrop ND-1000 (NanoDrop Technologies, Montchanin, DE, USA). mRNA were reverstranscripted by RevertAid™ H Minus First Strand cDNA Synthesis Kit (Thermo Scientific, Waltham, MA). 1 µg of total RNA was mixed oligo (dT)_18_ primer and denatured. Subsequently, the RNA sample was mixed with 5X reaction buffer, dNTP, RiboLock™ RNase Inhibitor, and RevertAid™ H Minus Reverse Transcriptase. The reverse transcription reaction was performed at 42^o^C for 60 min and terminated at 70^o^C for 5 min. The reverse transcription product was directly used in PCR amplification or stored at −20^o^C.

### Real Time PCR

The gene-specific primers for real time PCR were designed by Beacon Designer 7 program (Premier Biosoft International, Palo Alto, CA, USA) and the sequences are listed in Table 1. Real-time quantitative PCR was performed using SYBR Green Supermix (BIO-RAD, Hercules, CA) for 40 amplification cycles in an iCycler iQ5™ Real-Time PCR Detection System (Bio-Rad Laboratories). Relative transcript quantities were calculated using the ΔΔCt (threshold cycle) method with GAPDH as the reference gene amplified from the samples. All reactions were run in triplicate.

**Table 1:** Primers used in qPCR.

Genes	primer sequence (5'→3')	Length	Tm	GC%
ATM	CCGTGATGACCTGAGACAAGATG	23	57.1	52
	CAAGAACACCACTTCGCTGAGAG	23	57.1	52
ATR	CACCACCAGACAGCCTACAATG	22	56.7	55
	CAGAGCCACTTTGCCCTTTCC	21	56.3	57
TP53	CCATCCTCACCATCATCACACTG	23	57.1	52
	CACAAACACGCACCTCAAAGC	21	54.4	52
GADD45A	TGAACGGTGATGGCATCTGAATG	23	55.3	48
	AGTGTAGGGAGTAACTGCTTGAG	23	55.3	48
GADD45B	TTGAACTTGGTTGGTCCTTGTCTG	24	55.7	46
	TCTATGCTTCCCATCTCGCTCTC	23	57.1	52
P21	TCCTTTCCCTTCAGTACCCTCTC	23	57.1	52
	CCTTCTTCTTGTGTGTCCCTTCC	23	57.1	52
CDK1	GTCCGCAACAGGGAAGAACAG	21	56.3	57
	CGAAAGCCAAGATAAGCAACTCC	23	55.3	48
CCNB1	CTGTTGGTTTCTGCTGGGTGTAG	23	57.1	52
	CGCCTGCCATGTTGATCTTCG	21	56.3	57
CCNB2	ACAAGTCCACTCCAAGTTTAGGC	23	55.3	48
	CCAAGAGCAGAGCAGTAATCCC	22	56.7	55
GAPDH	CAAGGTCATCCATGACAACTTTG	23	43.5	60
	GTCCACCACCCTGTTGCTGTAG	22	59.1	63

### Protein Extraction and Western Blotting

After MIR exposure for 48 h, total protein was extracted using the Trizol reagent (Invitrogen) following the manufacturer’s instructions. Protein was solubilized in lysis buffer (7 M urea (Boehringer, Mannheim, Germany), 2 M thiourea, 4% CHAPS (J. T. Baker, Phillipsburg, NJ, USA) and 0.002% bromophenol blue (Amersco, Solon, OH, USA)) and the protein concentration was determined by BCA method (Pierce Biotech Inc., Rockford, IL, USA). Samples (30 µg) were mixed with sodium dodecyl sulfate-polyacrylamide gel electrophoresis (SDS-PAGE) sample buffer, boiled for 5 minutes, electrophoresed on a 10% SDS polyacrylamide gel, and electroblotted onto polyvinylidene difluoride (PVDF) membrane (Millipore). The membrane was blocked in 5% non-fat milk in TBST for 1 hour with gentle agitation. The membrane was incubated with the following primary antibody diluted in blocking buffer at 4^o^C overnight: rabbit anti-CDC25C (GeneTex, San Antonio, TX, USA; 1∶2000), rabbit anti-phospho-CDK1 (Thr161, Santa Cruz, CA, USA; 1∶100), mouse anti-CDK1 (Santa Cruz; 1∶100), rabbit anti-Cyclin B1 (GeneTex; 1∶500). To detect Actin, the membrane was stripped, blocked, and incubated with mouse anti-Actin (Millipore; 1∶1000) in blocking buffer for 1 hour at room temperature. After washing, the membrane was incubated with appropriate horseradish peroxidase-labeled secondary antibody (Sigma-Aldrich; 1∶100000) for 2 hours at room temperature. Signal was developed with ECL detection reagent (Millipore) and exposed to Fuji medical X-ray film.

### Cell Cycle Analysis

Cells from six independent experiments were collected, fixed in 70% ethanol, and stored at −20°C. Cells were then washed twice with PBS, resuspended in PBS containing 1 mg/mL RNase A and incubated at 37°C for 45 minutes and followed by propidium iodide (PI, 10 µg/mL) staining for 15 minutes. The DNA content of cells was then analyzed with a FACSCanto instrument (BD Biosciences Immunocytometry Systems, San Jose, CA, USA). The percentage of cells in different phases of the cell cycle was calculated by MultiCycle (DeNovo software).

### Statistics Analysis

Results are expressed as mean ± SD. The Student’s *t* test was used for analysis of the cell viability assay and real-time PCR data.

## Supporting Information

Figure S1 **MIR exposed medium does not affect the cell proliferation of A549.** A549 cells were seeded in 12-well plates overnight and then seeding medium was replaced with IR-exposed or unexposed (control) medium. Cell proliferation was determined by MTT assay after a 48-h exposure. The data are presented as mean ± SD from three independent experiments.
(TIF)

Figure S2 **The cell viability and morphology of MRC-5 cells were not affected by 48-hour exposure to MIR.** (A) Proliferation of MRC-5 cells was determined by an MTT assay as described in materials and methods. The data are presented as mean ± SD from three independent experiments. (B) Cell morphology was observed by phase-contrast microscopy. Scale bar represents 50 µm.
(TIF)

Figure S3 **Effect of MIR exposure on DNA double strain breaks in A549 cells.** Cells were seeded onto the glass coverslip in 12-well plate, exposure by MIR for 48 hours, fixed for staining and visualized by fluorescence microscopy. (A) 53BP1 was labeled with anti-53BP1 antibody and corresponded FITC– conjugated secondary antibody (green), and nuclei were stained with DAPI (blue). (B) γ-H2AX was labeled with anti-γ-H2AX antibody following corresponded FITC–conjugated secondary antibody (green) and nuclei were labeled with DAPI (blue). Scale bar represents 10 µm.
(TIF)
