# Supplementary figures and images for: Middle Infrared Radiation Induces G2/M Cell Cycle Arrest in A549 Lung Cancer Cells
Source: PLoS One. 2013 Jan 15;8(1):e54117. doi: 10.1371/journal.pone.0054117 (PMC3546001; doi:10.1371/journal.pone.0054117)

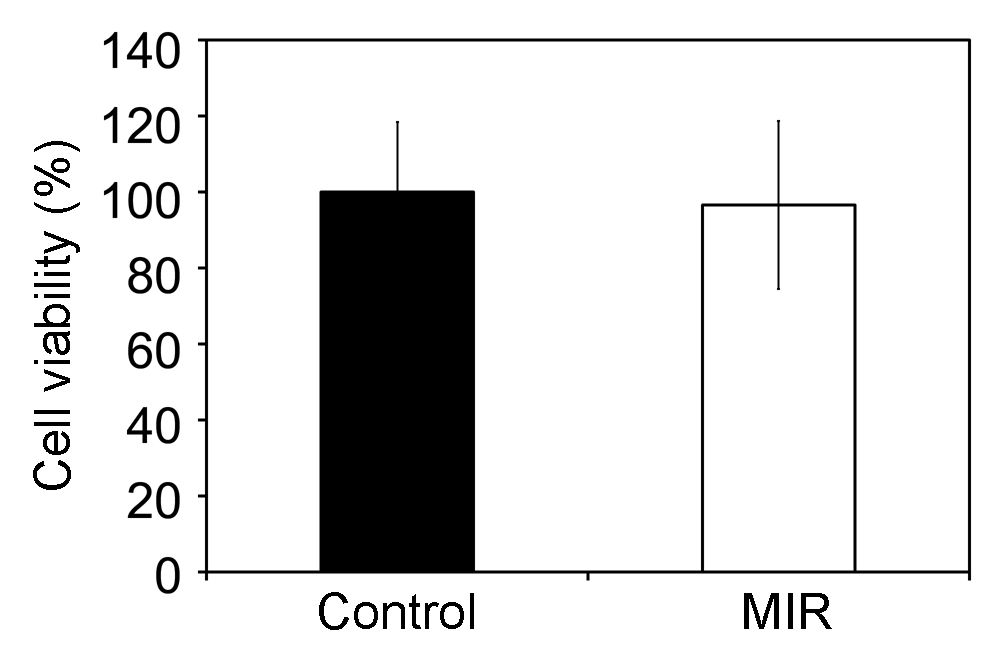

Supplement: Figure S1 — MIR exposed medium does not affect the cell proliferation of A549. A549 cells were seeded in 12-well plates overnight and then seeding medium was replaced with IR-exposed or unexposed (control) medium. Cell proliferation was determined by MTT assay after a 48-h exposure. The data are presented as mean ± SD from three independent experiments. (TIF) [file pone.0054117.s001.tif]

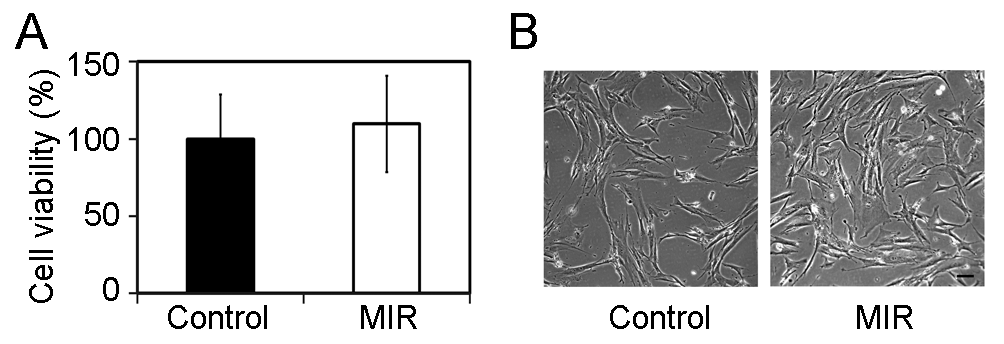

Supplement: Figure S2 — The cell viability and morphology of MRC-5 cells were not affected by 48-hour exposure to MIR. (A) Proliferation of MRC-5 cells was determined by an MTT assay as described in materials and methods. The data are presented as mean ± SD from three independent experiments. (B) Cell morphology was observed by phase-contrast microscopy. Scale bar represents 50 µm. (TIF) [file pone.0054117.s002.tif]

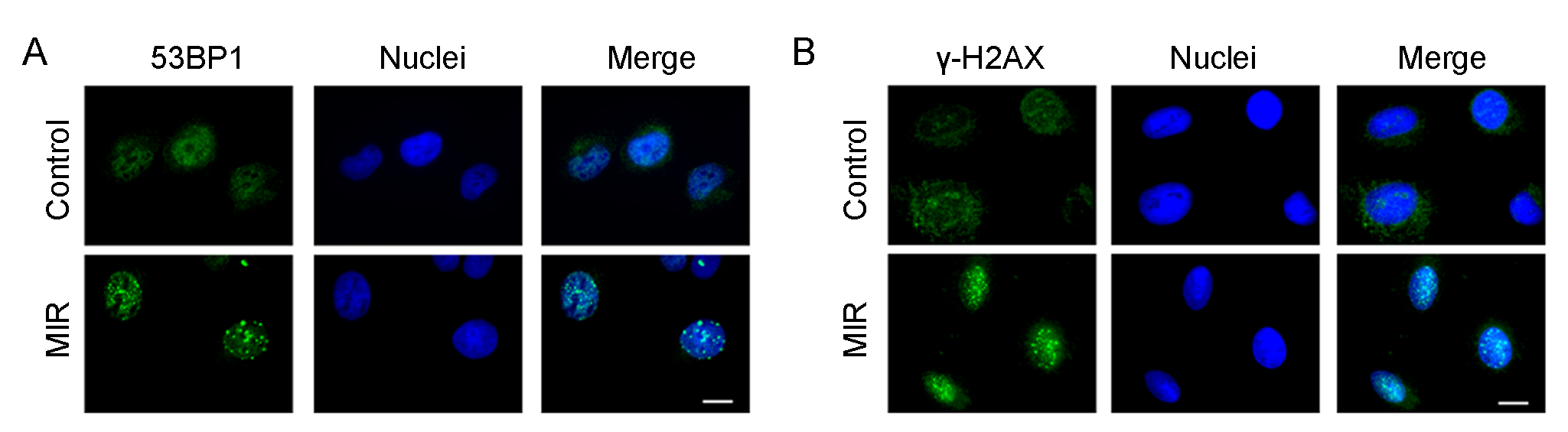

Supplement: Figure S3 — Effect of MIR exposure on DNA double strain breaks in A549 cells. Cells were seeded onto the glass coverslip in 12-well plate, exposure by MIR for 48 hours, fixed for staining and visualized by fluorescence microscopy. (A) 53BP1 was labeled with anti-53BP1 antibody and corresponded FITC– conjugated secondary antibody (green), and nuclei were stained with DAPI (blue). (B) γ-H2AX was labeled with anti-γ-H2AX antibody following corresponded FITC–conjugated secondary antibody (green) and nuclei were labeled with DAPI (blue). Scale bar represents 10 µm. (TIF) [file pone.0054117.s003.tif]
